# Futuristic electron transport layer based on multifunctional interactions of ZnO/TCNE for stable inverted organic solar cells†

**DOI:** 10.1039/d0ra08093d

**Published:** 2020-11-20

**Authors:** Md. Aatif, J. P. Tiwari

**Affiliations:** Advanced Materials and Devices Metrology Division (Photovoltaic Metrology Group), CSIR-National Physical Laboratory New Delhi 110012 India jai_ti2002@yahoo.com tiwarijp@nplindia.org +91-11-4560-8640; Academy of Scientific and Innovative Research (AcSIR) CSIR-HRDC Campus Ghaziabad 201002 India

## Abstract

Solution-processed inverted bulk heterojunction (BHJ) organic solar cells (OSCs) are expected to play a significant role in the future of large-area flexible devices and printed electronics. In order to catch the potential of this inverted BHJ technology for use in devices, a solar cell typically requires low-resistance ohmic contact between the photoactive layers and metal electrodes, since it not only boosts performance but also protects the unstable conducting polymer-based active layer from degradation in the working environment. Interfacial engineering delivers a powerful approach to enhance the efficiency and stability of OSCs. In this study, we demonstrated the surface passivation of the ZnO electron transport layer (ETL) by an ultrathin layer of tetracyanoethylene (TCNE). We show that the TCNE film could provide a uniform and intimate interfacial contact between the ZnO and photo-active layer, simultaneously reducing the recombination of electron and holes and series resistance at the contact interface. After successful insertion of TCNE between the ZnO film and the active layer, the parameters, such as short circuit current density (*J*_sc_) and fill factor (FF), greatly improved, and also a high-power conversion efficiency (PCE) of ∼8.59% was achieved, which is ∼15% more than that of the reference devices without a TCNE layer. The devices fabricated were based on a poly[[4,8-bis[(2-ethylhexyl)oxy]benzo[1,2-*b* : 4,5-*b*′]dithiophene-2,6-diyl]-[3-fluoro-2[(2-ethylhexyl)-carbonyl]-thieno[3,4-*b*]thiophenediyl]] (PTB7):(6,6)-phenyl C71 butyric acid methyl ester (PC71BM) blend system. These results suggest that this surface modification strategy could be readily extended in developing large-scale roll-to-roll fabrication of OSCs.

## Abstract 2

Solution-processed inverted bulk heterojunction (BHJ) organic solar cells (OSCs) are expected to play a significant role in the future of large-area flexible devices and printed electronics.

## Graphical abstract

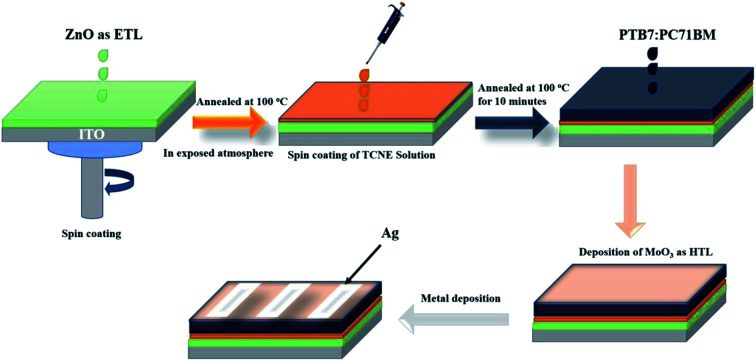

## 1. Introduction

Bulk-heterojunction (BHJ) organic solar cells (OSCs) have attracted significant consideration in the past few years due to their promising potential to provide environmentally safe, flexible, lightweight, and cost-effective solar cells. To date, power conversion efficiencies (PCEs) beyond ∼17% have rapidly developed for single-junction as well as tandem OSCs.^1,2^ However, their short life span, dreadful stability, and a relatively low power conversion efficiency (PCE) compared to inorganic solar cells have been the main limitations of their commercialization.

Generally, it is well known that, for a regular OSC, a blended active layer of bulk heterojunction (BHJ) is sandwiched between two different electrodes (*i.e.*, anode and cathode) with suitable interfacial layers. As per the direction of charge carriers, OSCs can be termed as conventional and inverted device configurations.^3^ In most of the conventional structure poly (3,4-ethylene dioxythiophene):poly (styrene sulfonate) (PEDOT:PSS) is used as anode interfacial layer/hole transporting layer (AIL/HTL) to adjust the ITO electrode. Nevertheless, conventional OSCs generally suffer from faster degradation and inadequate lifetime due to the hygroscopic and acidic behaviour of PEDOT:PSS and air sensitivity of Al cathode.^4^ As an alternative, BHJ OSCs with inverted configuration is a better solution that displays better efficiencies and extended lifetime than that of conventional counterparts. It allows the use of low air-sensitive high work function metals such as Ag and Au. The top anode electrodes and the transparent ITO used as a cathode are modified by interfacial layers with low work function, acting as an electron transport layer (ETL).^5^ The ETL plays an influential role in extracting and transporting photogenerated charge carriers from the photo-active layer to the cathode or anode. At the same time, the ETL also modifies the interface between the photo-active layer and the electrode, minimizing interface defects, and charge recombination.^6^ To realize a perfect ETL for enhancing the performance of OSCs, numerous materials including n-type metal oxide semiconductors such as titanium oxide (TiO_*x*_),^7,8^ Zinc Oxide (ZnO),^9^ stannic oxide (SnO_2_),^10^ cesium carbonate (Cs_2_CO_3_),^11,12^ and polymers, carbon-based materials, small-molecules,^13,14^ hybrids/composites,^15^ and other evolving contestants have been grown-up as ETL in inverted BHJ OSCs.^16–18^

Among them, ZnO is a more attractive material owing to its excellent optical transparency, relatively high electron mobility, environment-friendly nature, and ease of fabrication.^19,20^ The energy levels of zinc oxide are about 4.3 eV (conduction band minimum) and 7.8 eV (valence band maximum). This energy band position enables ZnO to play a significant role in electron collection and hole blocking.^21^ A variety of low-temperature and solution-based fabrication methods have been demonstrated to deposit ZnO as ETL.^22^ Despite the advantages of ZnO as ETL, surficial defects on ZnO thin-film can behave as recombination centers for photogenerated charge carriers, causing significant harm in both photovoltage and photocurrent, thus worsening the performance of devices.^23,24^ Additionally, they create adsorption sites for environmental oxygen and water molecules, which are highly detrimental to device stability.^25,26^ Consequently, the surface defect passivation strategy of ZnO has become vital concurrently in enhancing the PCE and the sustainability of inverted OSCs.

The most widely followed passivation methodologies are the insertion of suitable interfacial modifiers, such as conjugated polyelectrolytes (CPEs),^27^ alcohol/water-soluble conjugated polymers,^28^ self-assembled monolayers (SAMs),^29^ small molecules,^30^ and ionic liquids (ILs),^27^ at the interface which improved the electronic coupling between ZnO/photo-active blend resulting enhanced device characteristics. Moreover, polar solvent, or plasma treatment of the ZnO film, before the deposition of the active layer, represent effective ways to address the surface defects related issues.^31^

Hence, a general and straightforward method that can passivate the defects present on ZnO thin film is highly desirable for further enhancement in the performance of inverted BHJ OSCs.

In this work, we propose that the solution-processed organic small molecule TCNE can effectively passivate the surface defects of ZnO based ETLs. Herein, we have demonstrated three different concentrations (0.5, 1 and 2 mg ml^−1^) of TCNE as surface modifier between ZnO and photoactive blend to facilitate efficient transport of negative charge carriers from photoactive layer to ZnO film in inverted BHJ OSCs based on PTB7:PC71BM system, which does not only improve the *J*_sc_ but also enhanced FF. Subsequently, this work reveals that the TCNE can be used as a valid interface modification material in inverted BHJ solar cells. To the best of our knowledge, very few low-temperature processed ZnO and passivated ZnO films are used for the OSCs. For the first time, TCNE was used as a passivating layer in organic solar cells. Thus, our study reports a new low-temperature processed ETLs that are suitable for fabricating high-performance flexible OSCs with good stability. Besides, TCNE not only reduced the traps of the ZnO films but also contribute to the extraction of charge carriers as it has a strong potential to accept the electrons, which leads the improved electron mobility (∼10^−3^) of the ZnO/TCNE system and provide a larger interface between ETL and the active layer, which is essential for enhancing the performance of the inverted organic solar cells. Furthermore, the results reconfirm the importance of TCNE passivating layer design, indicating the great significance of this simple and effective approach for advancing the efficiency of iOSCs. It could be a promising alternative for other optoelectronic devices, which is beneficial for large-scale production.

## 2. Experimental

### 2.1 Materials

Indium tin oxide (ITO)-coated glass sheet (10–15 Ω sq^−1^) was purchased from Lumtech Ltd. and used as substrates and electrode for all the devices. Zinc acetate dihydrate (99.9%), potassium hydroxide (KOH), tetracyanoethylene (TCNE), 1,2-dichlorobenzene (DCB, anhydrous, 99%), chlorobenzene (anhydrous CB, 99.8%), and anhydrous methanol were obtained from Sigma-Aldrich. Chloroform and *n*-butanol (>99%) were procured from Alfa Aesar. One-Material Inc provided the donor polymer PTB7 and electron acceptor PC71BM (99.5%). All the above materials and solvents have been utilized as received without any further purification.

### 2.2 Preparation of ZnO nanoparticles (NPs) and TCNE solutions

The transparent solution of ZnO nanoparticles was prepared by literature procedures.^32,33^ KOH (1.48 g, 23 mmol) solution in 65 ml of methanol was added dropwise to a round bottom flask containing zinc acetate dihydrate (2.95 g, 13.4 mmol) in 125 ml of methanol solution, within 15–20 minutes at 65 °C under magnetic stirring for 2.5 hours. Then, the solution was cool down to decant the supernatant, and the solid white precipitate was washed twice with methanol, and the suspension was then centrifuged to collect the nanoparticles. Afterward, *n*-butanol and chloroform were used to disperse the precipitate, and a ZnO NPs solution with the desired concentration of 15 mg ml^−1^ was obtained. Before use, the ZnO NPs solution was then filtered through a 0.45 μm PTFE syringe filter. We can use this ZnO NPs solution up to several months later.

The TCNE solutions were then prepared by varying different concentrations up to 2 mg ml^−1^ (*i.e.*, 0.5, 1, and 2 mg ml^−1^) in anhydrous methanol. The uniform solutions were formed with constant stirring at room temperature for 3 hours. Henceforward, these solutions were then used to spun the TCNE onto the ZnO ETL layer before further steps.

### 2.3 Measurements and characterisation

The Shimadzu UV-2401 PC spectrophotometer was used to carry out the absorption and transmittance spectra of ZnO and ZnO/TCNE films on the glass/ITO substrates. The surface roughness of ZnO and ZnO/TCNE films surface were evaluated by the atomic force microscope (Nano First-3100) in a tapping mode. XRD (X-ray diffraction) patterns are taken using a Rigaku X-ray diffractometer equipped with CuKα (1.54 Å). Morphological analysis of prepared samples is done using Field Emission Scanning Electron Microscope (FE-SEM, Zeiss-Ultra Plus Gemini Co.). SEM system is also used to obtain cross-sectional images. The photoluminescence (PL) studies of glass/ITO/ZnO/PTB7:PC71BM and glass/ITO/ZnO/TCNE/PTB7:PC71BM films were recorded with an excitation wavelength of 500 nm, Jasco FP-6500 spectrophotometer. Efforts to measure the exact thickness of the TCNE layers gave unreliable results because the TCNE layers were too thin.

### 2.4 Photovoltaic characterization

The computer-programmed Keithley 2420 was employed for characterizing the device performance of all iOSCs, in the dark or under the illumination of 100 mW cm^−2^ (AM 1.5 G) using a solar simulator. Pyranometer was used to adjust the intensity of incident light. All the devices were then tested with a scan rate of 0.2 V s^−1^, the delay period was 10 ms, and the scan step was 0.02 V in the reserve direction from −1 to 1 V. Further, the measurement for electron-only devices was also done with the Keithley 2420 equipment, under dark condition.

### 2.5 Device fabrication

**Fig. 1:**
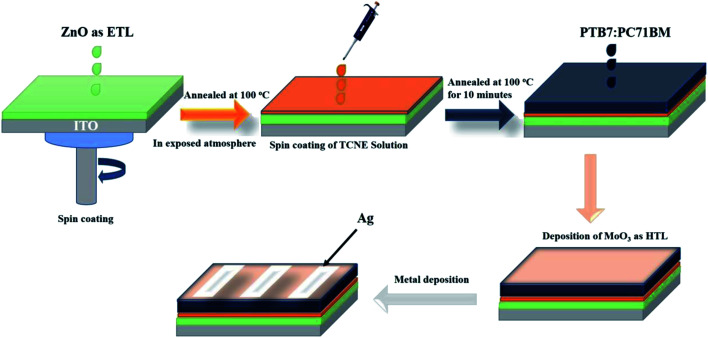
Schematic representation of organic solar cell fabrication.

The schematic depiction of complete device fabrication is shown in Fig. 1. The fabricated device structure was inverted as ITO/ZnO/PTB7:PC71BM/MoO_3_/Ag and ITO/ZnO/TCNE/PTB7:PC71BM/MoO_3_/Ag for reference and passivated cells. Firstly, the ITO-coated glass substrates were etched using a laser scriber system installed in the solar energy cleanroom complex (SECRC). The pre-patterned ITO substrates were cleaned by sequentially with soap solution, deionized water, acetone, and isopropanol (IPA) 30 minutes each in an ultrasonic cleaner bath, followed by drying with nitrogen and kept inside the vacuum oven at 120 °C for 15 minutes. Subsequently, the dried ITO substrates were then moved to the ultraviolet–ozone chamber for 15 min to remove the organic residues on the surface of the substrates and make it hydrophilic. Now the ZnO NPs solution with a concentration of 15 mg ml^−1^ in *n*-butanol and chloroform was coated on ITO substrates at the spin frequency of 2500 rpm for 45 s and annealed at 100 °C for 10 minutes in the exposed atmosphere. The thickness of the ZnO ETL film is ∼30 nm. All the ZnO coated ITO substrates were then transferred to a nitrogen-filled MBRAUN glovebox system for further processing. Afterward, the TCNE solution in methanol with various concentrations (*i.e.*, 0.5, 1, and 2 mg ml^−1^) was then spin-cast at 4000 rpm for 45 s on ZnO film and annealed at 100 °C for 10 minutes inside the glove box. The thickness of the TCNE layer on the ZnO film is too thin to measure. The BHJ composition of PTB7:PC71BM blend system (1 : 1.5 wt%, 25 mg ml^−1^) was weighed in anhydrous chlorobenzene (CB)^32^ and kept on stirrer at 60 °C for at least of 12 h. After being left to be stirred at 60 °C overnight, DIO (30 μL) was added to the mixture and was stirred at 60 °C under dark conditions for 1 h before use. Now the PTB7:PC71BM BHJ layers deposited on freshly prepared ZnO and ZnO/TCNE films at 1500 rpm for 60 s inside the glovebox and kept it in a vacuum for over the night to slow evaporation of the solvent. The thickness of the active layer was ∼120 nm. Next, these samples were then transferred to a vacuum chamber in the nitrogen-filled glovebox system, where MoO_3_ (10 nm) and Ag (100 nm) sequentially thermally deposited on the top of BHJ layers using a shadow mask in a vacuum of 2 × 10^−6^ torr. The pixel area of the devices was 0.06 cm^2^.

To further scrutinize the charge transport, devices were fabricated with the configuration of ITO/ETLs/PTB7:PC71BM/LiF/Al. To evaluate the charge extraction efficiency and space charge limited conduction (SCLC), electron only devices were then fabricated on ITO substrates which were cleaned consecutively by using a soap solution, deionized water, acetone, and isopropanol (IPA) for 15 min each in ultra-sonicator, afterward, all the ITO substrates kept at 120 °C for 15 min in a vacuum oven which is then followed by UV–O_3_ treatment for 15 min. To fabricate the electron-only devices, ETLs ZnO and ZnO/TCNE spin-casted on ITO as in the section of device fabrication. The blend of PTB7:PC71BM spin-coated with varying frequency rate and annealed at 70 °C for 10 min. To elude the inbuilt potential in devices, the LiF layer was then thermally deposited on the top of PTB7:PC71BM films. Finally, a 100 nm thick Al electrode was deposited in the evaporating chamber (∼10^−6^ torr) with a defined active area of 0.06 cm^2^.

## 3. Results and discussion

**Fig. 2:**
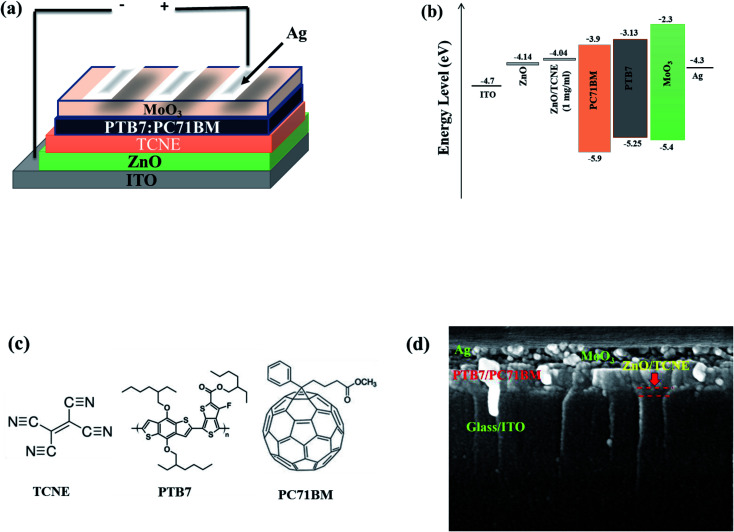
(a) Inverted BHJ organic solar cells with TCNE-coated ZnO. (b) Energy level diagram of the materials used in OSC device (c) chemical structures of TCNE (surface passivator), PTB7 (donor), and PC71BM (acceptor) (d) SEM image for the cross-section of the device.

The molecular structures of PTB7, PC71BM, and TCNE as the donor, acceptor, and passivator materials used in our work and a simple schematic illustration of the device architecture and SEM cross-section image of a fabricated device are shown in Fig. 2. The preparation of ZnO and ZnO/TCNE is explained earlier in the experimental section. The ZnO/TCNE ETL has significantly given enhanced performance compared to that of the reference device. The enhanced results were then confirmed with a detailed investigation using structural, morphological, and optoelectronic analysis of the ZnO/TCNE and compared to that of the regular ZnO. TCNE-induced changes in the performance of inverted BHJ OSC (Table 1).

**Table 1:** An exhaustive review of enhancement in PCE of inverted BHJ OSCs using ZnO/passivated ZnO as ETL based on the P3HT:PC60BM, PTB7:PC71BM, and PTB7-Th:PC71BM, *etc*

Device design	ETL type	PCE [%] improvement	*V*_oc_ [V]	*J*_sc_ [mA cm^−2^]	FF [%]	Ref.
ITO/ETL/PTB7-Th:PC71BM/MoO_3_/Ag	ZnO → ZTO	8.46 → 9.02	0.80	17.61	64.39	1
ITO/ETL/P3HT:PC61BM/MoO_3_/Ag	ZnO → ZnO/CaF_2_	3.21 → 4.22	0.63	10.51	64.18	2
ITO/ETL/PBDT-TNT:PC71BM/MoO_3_/Al	ZnO → ZnO/PFN-Br	6.1 → 8.40	0.75	17.40	61.0	3
ITO/ETL/P3HT:PC61BM/PEDOT:PSS/Ag	ZnO → ZnO/C-PCBSD	3.5 → 4.40	0.60	12.80	58.0	4
ITO/ETL/P3HT:ICBA/PEDOT:PSS/Ag	ZnO → ZnO/C-PCBSD	4.81 → 6.22	0.84	12.40	60.0	4
ITO/ETL/PBDT-DTBT:PC71BM/MoO_3_/Al	ZnO → ZnO/PC60BM-G2	4.77 → 6.42	0.73	14.00	62.0	5
ITO/ETL/PTB7:PC71BM/MoO_3_/Ag	ZnO → ZnO/PFN	7.28 → 8.01	0.75	15.50	68.9	6
ITO/ETL/P3HT:PC61BM/MoO_3_/Al	ZnO → ZnO/Cs_2_CO_3_	3.74 → 4.26	0.58	11.27	65.20	7
ITO/ETL/P3HT:PC61BM/MoO_3_/Ag	ZnO → ZnO/DNA	3.43 → 4.09	0.58	11.86	58.71	8
ITO/ETL/P3HT:PC61BM/MoO_3_/Ag	ZnO → ZnO/PEIE	4.01 → 4.07	0.62	10.61	62.0	9
ITO/ETL/PBTB-T:ITIC/MoO_3_/Ag	ZnO → ZnO/APTES	9.6 → 10.2	0.88	17.65	65.10	10
ITO/ETL/PTB7:PC71BM/MoO_3_/Ag	ZnO → ZnO/PTMAHT	7.28 → 7.64	0.74	15.3	67.5	11
ITO/ETL/PTB7:PC71BM/MoO_3_/Ag	ZnO → ZnO/[BMIM]BF_4_	8.94 → 9.56	0.78	17.70	73.5	12
ITO/ETL/PTB7:PC71BM/MoO_3_/Ag	ZnO → ZnO/PEI	6.99 → 8.76	0.73	17.19	69.6	13
ITO/ETL/PTB7-F20:PC71BM/PEDOT:PSS + Au NPs/Ag	ZnO → ZnO/ripple/ALD-ZnO	6.99 → 7.92	0.68	17.24	67.2	14
ITO/ETL/PIDTT-DFBT-TT:PC71BM/GO/MoO_3_/Ag	ZnO → ZnO/PCBM COOH	3.08 → 7.29	0.97	11.6	64.5	15
ITO/ETL/PBDBT-IT:M/MoO_3_/Ag	ZnO → ZnO/PBD	10.8 → 11.6	0.93	16.7	74.1	16
ITO/ETL/PCE-10:PC71BM/MoO_3_/Ag	ZnO → ZnO/NE	7.94 → 9.41	0.79	17.65	67.45	17
ITO/ETL/PCE-10:IEICO-4F/MoO_3_/Ag	ZnO → ZnO/C_60_-SAM	9.46 → 10.0	0.71	22.92	61.18	18
ITO/ETL/PTB7-Th:PC71BM/MoO_3_/Ag	ZnO → ZnO/Ba(OH)_2_	7.12 → 8.54	0.81	15.34	68.20	19
ITO/ETL/PTB7-Th:PC71BM/MoO_3_/Al	ZnO → ZnO/PEO	8.42 → 9.57	0.80	17.4	68.60	20
ITO/ETL/PTB7-Th:PC71BM/MoO_3_/Ag	ZnO → ZnO/l-Arg	8.09 → 9.31	0.78	17.49	68.22	21
ITO/ETL/PTB7-Th:PC71BM/MoO_3_/Ag	ZnO → ZnO/NS4	9.16 → 9.92	0.79	17.3	73.7	22
ITO/ETL/PTB7-Th:PC71BM/MoO_3_/Ag	ZnO → ZnO/PEOz	8.81 → 9.57	0.80	17.21	68.97	23
ITO/ETL/PTB7:PC71BM/MoO_3_/Ag	ZnO → ZnO/CsSt	6.97 → 8.46	0.73	17.07	69.1	24
ITO/ETL/PTB7:PC71BM/MoO_3_/Ag	ZnO → r-GO/ZnO/TiO_2_	7.57 → 8.61	0.75	17.67	65.0	25
ITO/ETL/PTB7:PC71BM/MoO_3_/Ag	ZnO → ZnO/S-CdS	6.8 → 8.0	0.74	16.19	66.6	26
**ITO/ETL(∼30 nm)/PTB7:PC71BM (∼120 nm)/MoO**_**3**_ **(∼10 nm)/Ag(∼100 nm)**	**ZnO → ZnO/TCNE**	**7.49 → 8.59**	**0.76**	**18.6**	**64.20**	**This work**

**Fig. 3:**
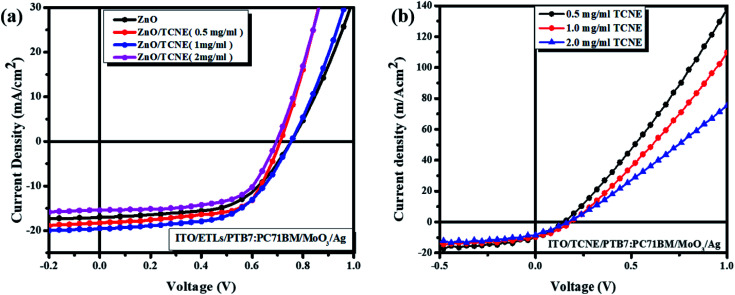
Current density–voltage (*J*–*V*) curves of the inverted BHJ devices (a) with ZnO and ZnO/TCNE with different concentrations as ETLs and (b) with TCNE only as ETLs with same concentrations.

The current density–voltage (*J*–*V*) curves of fabricated inverted BHJ OSCs based on PTB7:PC71BM with and without TCNE passivator on the top of ZnO ETL was done under 1.5 G, 100 mW cm^−2^ illumination portrayed in Fig. 3a and b. Moreover, the corresponding photovoltaic parameters are summarized in Tables 2 and 3. The schematic depiction of fabricated device structure and molecular structures of the polymer donor, fullerene acceptor, and TCNE materials are presented in Fig. 2. Further, we optimized the device performance by optimizing the TCNE molecule concentration as 0.5 mg ml^−1^, 1 mg ml^−1^, and 2 mg ml^−1^ in methanol. From Table 2, one may observe that the considerable effects of ZnO/TCNE on their device performance. The reference devices with ZnO as ETL shows the best PCE of 7.47% with *V*_oc_ = 0.73, *J*_sc_ = 16.2, and FF = 57.6%. Interestingly, the incorporation of ZnO/TCNE in iOSCs showed a great enhancement compared with the reference device at a minimal amount of TCNE (0.5 and 1 mg ml^−1^). However, further increasing the concentration of TCNE from 1 mg ml^−1^ to 2 mg ml^−1^, the devices show an abrupt decline in their performance parameters, even poorer than that of ZnO only. The low *J*_sc_ and poor FF may be attributed to a small shunt resistance and high series resistance because of an overdue amount of TCNE material at a higher concentration, such as 2 mg ml^−1^. The iOSCs using ZnO/TCNE (0.5 mg ml^−1^), and ZnO/TCNE (1 mg ml^−1^) and ZnO/TCNE (2 mg ml^−1^) as ETLs demonstrate the best photovoltaic parameters as *V*_oc_ = 0.72, *J*_sc_ = 17.8, FF = 59.6%, *V*_oc_ = 0.76, *J*_sc_ = 18.6, FF = 64.2%, and *V*_oc_ = 0.70, *J*_sc_ = 14.8, FF = 54.6%, respectively, with a PCE of 7.90%, 8.59%, and 6.59%. The improvements of *J*_sc_ and *V*_oc_ are mainly due to a significant modification of work function (WF), even though the suppression of charge recombination could be another point.^34^ On the other hand, the enhancements in FF may originate from the surface passivation, which decreases oxygen vacancy (V_O_) related to traps in ZnO with the incorporation of TCNE. Besides, the devices with the structure of ITO/TCNE/PTB7:PC71BM/MoO_3_/Ag with the same optimized concentration were also fabricated and showed inferior results, which is shown in Table 3. To determine the reasons for the improved performance on the ZnO/TCNE ETL-based devices, a series of measurements on ZnO and ZnO/TCNE films were carried out.

**Table 2:** Photovoltaic parameters of the OSCs based on ITO/ETLs/PTB7:PC71BM/MoO_3_/Ag inverted device architectures under the illumination of AM 1.5 G, 100 mW cm^−2^^a^

ETL type	*V*_oc_ [V]	*J*_sc_ [mA cm^−2^]	FF [%]	PCE [%]
Pristine ZnO	0.73 ± 0.002	16.2 ± 0.18	57.6 ± 0.33	7.47 ± 0.22
ZnO/**TCNE** (**0.5 mg ml**^**−1**^)	0.72 ± 0.003	17.8 ± 0.21	59.6 ± 0.11	7.90 ± 0.25
ZnO/**TCNE** (1 **mg ml**^**−1**^)	0.76 ± 0.00	18.6 ± 0.05	64.2 ± 0.09	8.59 ± 0.19
ZnO/**TCNE** (2 **mg ml**^**−1**^)	0.70 ± 0.002	14.8 ± 0.19	54.6 ± 0.32	6.59 ± 0.26

a Average device parameters shown were calculated from over 12 independent inverted organic solar cells. All the device testing was carried out at room temperature and in an open atmosphere.

**Table 3:** Photovoltaic parameters of the OSCs based on ITO/TCNE/PTB7:PC71BM/MoO_3_/Ag inverted device architectures under the illumination of AM 1.5 G, 100 mW cm^−2^

Pristine TCNE as ETL with different conc. in methanol	*V*_oc_ [V]	*J*_sc_ [mA cm^−2^]	FF [%]	PCE [%]
0.5 mg ml^−1^	0.12	9.50	32.9	0.46
1 mg ml^−1^	0.16	9.78	37.4	0.58
2 mg ml^−1^	0.16	8.46	31.6	0.42

### 3.1 Optical investigations of ZnO and TCNE modified-ZnO films

**Fig. 4:**
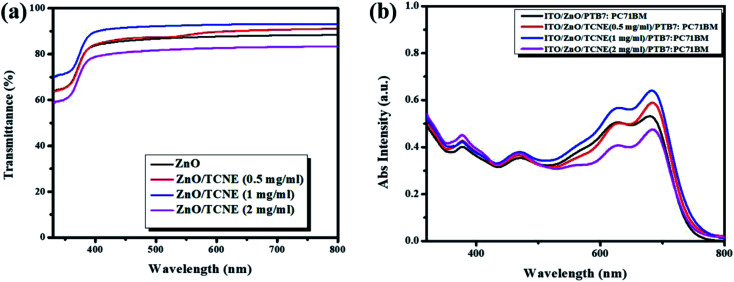
(a) Optical transmission of ZnO film and different concentrations of TCNE solution coated on ZnO, (b) corresponding absorption spectra with PTB7:PC71BM BHJ system.

To evaluate the function of the ZnO and TCNE modified ZnO ETLs layer, we have measured the optical transmission spectra of ETLs to determine the overall performance of iOSCs. Generally speaking, the efficient penetration of photons, transparency of ETLs are needed to be over 85% (ref. 35) in the range of visible wavelength, which ranges from 400 to 800 nm. The increase in the transmittance can significantly affect the photocurrent generated by the active layer, resulting in a higher PCE of iOSCs. Fig. 4a display the transmission spectra of ZnO and ZnO/TCNE (TCNE as 0.5 mg ml^−1^, 1 mg ml^−1^, and 2 mg ml^−1^) ETLs deposited on glass/ITO substrates. It can be noted that the ZnO/TCNE (1 mg ml^−1^) showed a wider range of transmission towards the UV region as compared to that of the pristine ZnO, 0.5 mg ml^−1^, and 2 mg ml^−1^ TCNE modified ZnO, indicating the absorbed photons by PTB7:PC71BM can generate charge carriers that can be transferred by the respective ETL and HTL layers. Also, these results are well-matched with the photovoltaic performance of PTB7:PC71BM BHJ solar cells. The excellent transmittance spectra of TCNE modified ZnO in the visible range is a shred of strong evidence that proves it a good alternate as ETLs for large-area manufacturing of iOSCs in coming future.

Fig. 4b shows the absorption spectra of ITO/PTB7:PC71BM and ITO/ETL/PTB7:PC71BM BHJ with different ETLs. These absorption plots are the combination of PTB7 and PC71BM films representing good mixing between the individual components. The spectra show the prominent absorption bands between 350–750 nm in the UV and visible region, attributed to the chain aggregations and π–π* electron transition of the conjugated chains. Moreover, the two significant peaks at 623 nm and 678 nm for PTB7 and the absorption range between 450–500 nm represents the characteristics peaks for PC71BM.^36^ It can be seen that the enhanced absorption intensity for ITO/ZnO/TCNE/PTB7:PC71BM, BHJ system is higher for the films consisting of ZnO/TCNE (1 mg ml^−1^) as an ETL. The enhancement of absorption of photons in the ETL ZnO/TCNE (1 mg ml^−1^) helps us to improve the PCE of iOSCs.

### 3.2 XRD analysis of pristine ZnO and TCNE passivated ZnO

**Fig. 5:**
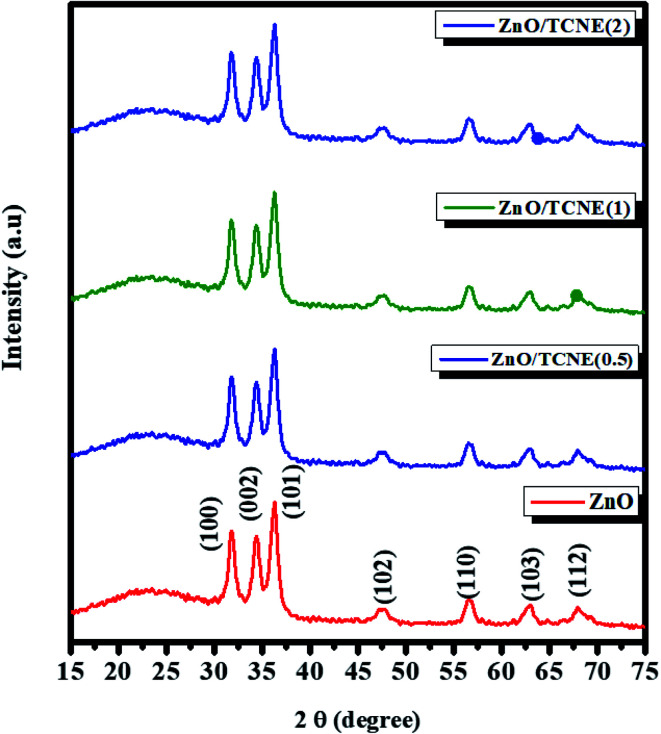
XRD pattern of pristine ZnO and ZnO/TCNE deposited on glass/ITO substrates.

The obtained XRD studied the crystallinity and the preferred crystal orientation of as-synthesized pristine ZnO and ZnO/TCNE films. The XRD patterns for pristine ZnO and ZnO/TCNE are shown in Fig. 5. For pristine ZnO, diffraction peaks observed around 31.7°, 34.49°, 36.28°, 47.48°, 56.78°, 63.12°, and 67.98° correspond to (100), (002), (101), (102), (110), (103) and (112) respectively, which agrees well with the reported data of pure hexagonal wurtzite phase of ZnO (JCPDS card no. 80-0075).^37,38^ All the samples exhibited similar diffraction peaks with no change in the relative intensity of the peaks. All diffraction peaks were adequately indexed. In the case of ZnO/TCNE, no other diffraction peak was observed with the addition of the TCNE passivation layer. TCNE has an amorphous character, and it provides surface passivation to the ZnO ETL film, henceforth enhance active layer and ETL electronic coupling, increases charge extraction from the active layer resulting in and improving the values of *J*_sc_ and FF.

### 3.3 Morphological investigation of ZnO and ZnO/TCNE films using AFM and SEM

**Fig. 6:**
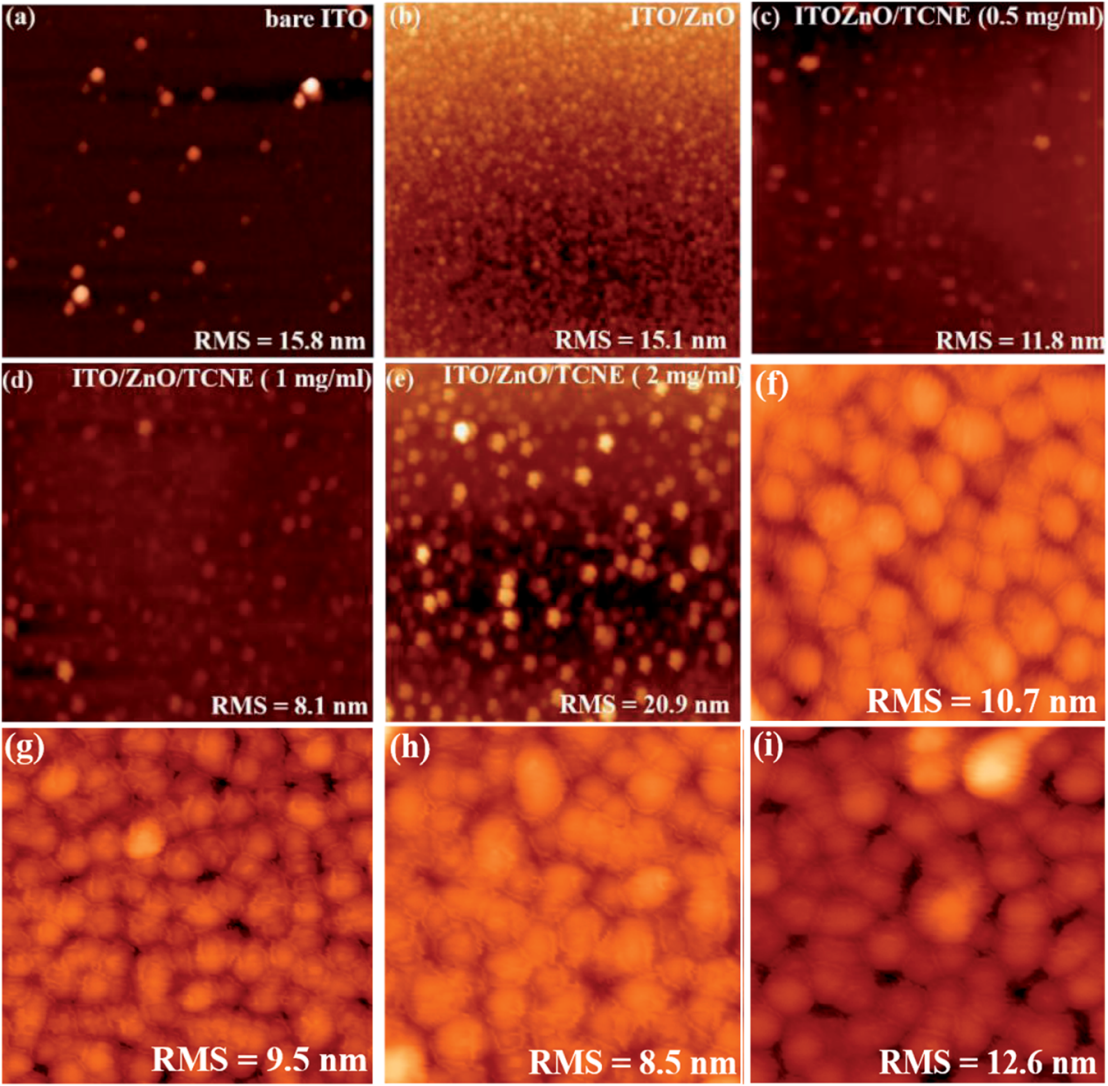
Atomic force microscopy (AFM) (10 × 10 μm^2^) surface images (a–e) showing RMS of bare ITO, pristine ZnO, and ZnO/TCNE (as 0.5 mg ml^−1^, 1 mg ml^−1^, and 2 mg ml^−1^ of TCNE) films on ITO glass substrates and (f–i) 5 × 5 μm^2^ AFM images of PTB7:PC71BM blend films deposited on ZnO and ZnO/TCNE films.

Apart from the optical properties of ZnO and ZnO/TCNE, the surface morphology is further a salient feature for evaluating the class of ETLs as it has a substantial impact on the device performance of inverted BHJ organic solar cells. The suitable surface roughness of ETLs used to provide a good interfacial contact with the active layer, allowing an efficient charge extraction and carrier transport at the interface from the active layer to ETL in iOSCs.^39^ We examined the changes in interfacial morphology and roughness of bare ITO, ZnO and ZnO passivated with varying quantity of TCNE (0.5, 1, and 2 mg ml^−1^), *via* the top-view SEM and AFM characterizations. Thus, atomic force microscopy (AFM) in tapping mode was used to clarify the surface roughness of ZnO and ZnO/TCNE, ETLs. Fig. 6a–i shows the AFM height images of films on the ITO glass substrates. The surface roughness root-mean-square (RMS) values of bare ITO, ZnO, ZnO/TCNE (0.5 mg ml^−1^), ZnO/TCNE (1 mg ml^−1^), and ZnO/TCNE (2 mg ml^−1^) are shown in Fig. 6a–e are 15.8, 15.1, 11.8, 8.1 and 20.9 nm, respectively. Compared to that of ZnO only, TCNE-modified ZnO samples showing slight changes in RMS surface roughness values. However, it is clearly shown that the homogenous and smooth surface, with 8.1 nm RMS value, was obtained for the ZnO/TCNE (1 mg ml^−1^) samples. In general, a lower RMS surface roughness value is needed to guarantee an intimate interfacial contact between the ETLs and the photo-active layers, contributing to reducing the series resistance (*R*_s_) of iOSCs to enhance the PCE.^40,41^

**Fig. 7:**
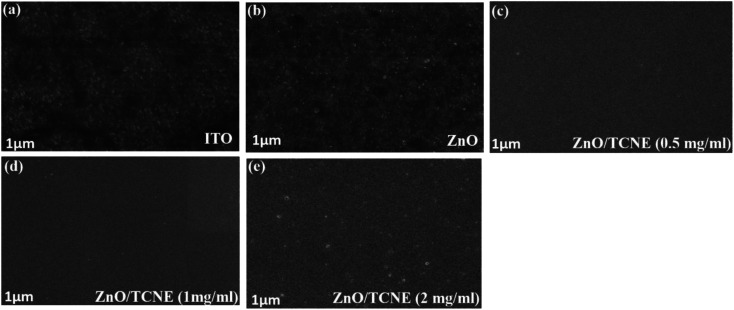
Top view SEM images of (a) bare ITO, (b) ZnO, (c–e) different TCNE concentrations (as 0.5 mg ml^−1^, 1 mg ml^−1^, and 2 mg ml^−1^) coated on ZnO film.

Additionally, a substantial change in the photoactive blend nanomorphology was observed when it was coated on the passivated ZnO.^42^ As shown in Fig. 6f–i, the PTB7:PC71BM blend exhibits a finer nanomorphology of the ETL consisting of TCNE with 1 mg ml^−1^ with the lowest RMS value of 8.5 nm. Fig. 7a–e present the top-view SEM images of naked ITO, ITO/ZnO, and ITO/ZnO/TCNE samples. One can undoubtedly detect that the ZnO and ZnO/TCNE films are uniform, dense, covering entire ITO substrates; this proposed the efficient UV-vis light absorption of ETLs and rejected possible short circuit of iOSC devices. It seems that the ZnO film, which is passivated by 1 mg ml^−1^ TCNE in methanol (Fig. 7d) has revealed the best morphology in comparison with other concentrations of TCNE, showed the iOSCs based on ZnO/TCNE (1 mg ml^−1^) might show the best device results. It has been pointed out that compared to the ZnO/TCNE (0.5 mg ml^−1^) or ZnO/TCNE (2 mg ml^−1^), the crystalline size of ZnO/TCNE (2 mg ml^−1^) was somewhat bigger, and this might occur due to an overdue quantity of TCNE (2 mg ml^−1^) on ZnO film (Fig. 7e). As is clear from Fig. 7d that the ZnO/TCNE (1 mg ml^−1^) layer has a denser surface morphology than a pristine layer, which is beneficial for achieving intimate contact between ZnO interfacial layer and the active layer.^43^

### 3.4 Charge collection and transport study

**Fig. 8:**
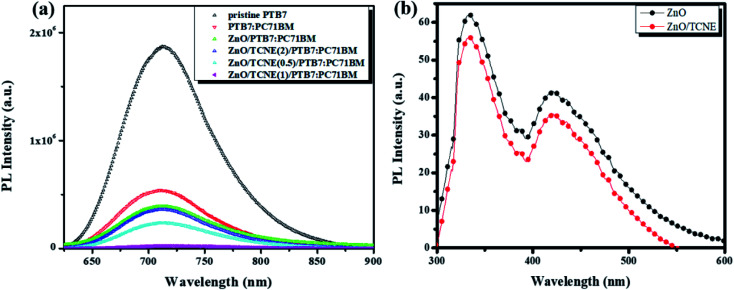
(a) The photoluminescence (PL) spectra of neat PTB7, PTB7:PC71BM, ZnO/PTB7:PC71BM, and ZnO/TCNE/PTB7:PC71BM at different concentrations of TCNE coated on ZnO films on ITO substrates (b) PL spectra of ZnO nanoparticle film with and without TCNE layer.

The photoluminescence (PL) investigations are a suitable tool to understand the charge transport/charge transfer kinetics and recombination in iOSCs.^44^ The dynamics of charge carriers at the ETLs/active layer interface is an essential criterion to determine the photovoltaic performance of the devices. In view of that, we performed the room temperature (RT) PL for the neat PTB7 film, PTB7:PC71BM blend film, and PTB7:PC71BM film on ZnO and ZnO/TCNE ETLs to explore the passivation effect of ZnO/TCNE film on the charge transfer and carrier extraction at the interface. Fig. 8a describes that the PL intensity of ZnO/TCNE/PTB7:PC71BM was significantly quenched in all wavelength ranges (500 to 900 nm) in comparison to the PTB7:PC71BM and ZnO/PTB7:PC71BM sample implying that ZnO/TCNE films are more pronounced in suppressing the carrier recombination while empowering the electron extraction and transport towards the cathodes. Among ZnO/TCNE/PTB7:PC71BM samples, it appears that the ZnO/TCNE (1 mg ml^−1^) shows the higher quenching between 680 to 800 nm, indicating the maximum suppression of charge recombination. These improvements in the quenching of PL denotes the absorption of higher photoinduced charge carriers, which are converted into excitons.^45,46^ Thus, the PL quenching in the ZnO/TCNE (1 mg ml^−1^) is in accordance with the enhanced device performance.

Also, PL under 300 nm photoexcitation for pristine ZnO and ZnO/TCNE film, also represented in Fig. 8b. The emission peak at 333 nm may be due to exciton emission.^47,48^ The shoulder at 423 nm may be instigating from the Zn interstitial defects or could be credited to transition among photoexcited carriers, surface defects, oxygen vacancies, *etc.*^4,49^ As evident from Fig. 8b, the intensity of emission shrinks for the ZnO/TCNE layer concerning the neat ZnO film, which may be a signal the tailoring of surface traps in the passivated layer. In most of the photovoltaic (PV) devices, the reduction of traps can reduce the trap-assisted interfacial recombination of charge carriers, reflecting the boost in short-circuit current density (*J*_sc_) as well as FF of the device; thus, the PCE of the device was improved.

**Fig. 9:**
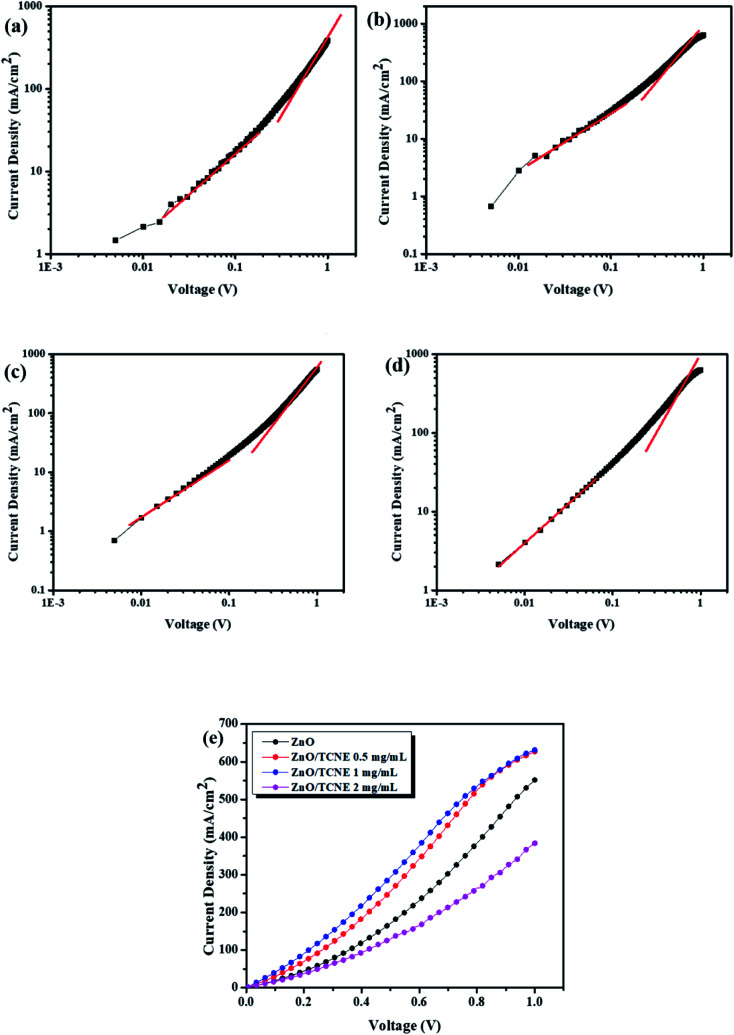
(a–d) Linear-fitting for *J*^0.5^–*V* and (e) *J*–*V* characteristics of the electron-only devices with ZnO and different TCNE concentrations coated on ZnO as ETLs, measured in the dark.

Finally, to gain more in-depth insight into the electron-transport properties of ZnO/TCNE films, the electron mobility of the devices has been investigated. Electron-only devices with the structure of ITO/ETLs/PTB7:PC71BM/LiF/Al were then fabricated. As shown in Fig. 9a–d, it tells that the current-density of the fabricated devices was declined drastically with the increased concentration of TCNE from 1 to 2 mg ml^−1^ (see Fig. 9d), and suggested an adverse effect on ZnO with an overdue amount of TCNE (2 mg ml^−1^). For measuring *μ*_e_ by space charge limited conduction (SCLC) method. The *μ*_e_ value is calculated by the Mott–Gurney equation,^50,51^1
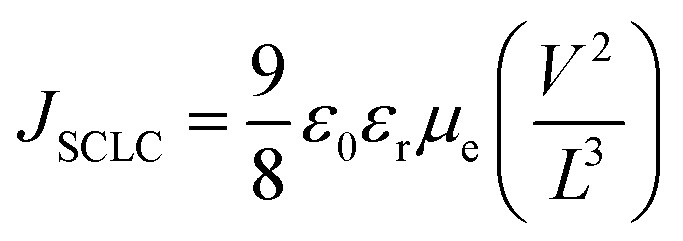


The *μ*_e_ was found to be 6.23 × 10^−4^ cm^2^ V^−1^ s^−1^, 1.28 × 10^−3^ cm^2^ V^−1^ s^−1^, 1.34 × 10^−3^ cm^2^ V^−1^ s^−1^ and 5.22 × 10^−4^ cm^2^ V^−1^ s^−1^for ZnO and ZnO/TCNE (0.5 mg ml^−1^), ZnO/TCNE (1 mg ml^−1^) and ZnO/TCNE (2 mg ml^−1^) respectively. It was observed that the *μ*_e_ of the electron-only devices with ZnO/TCNE (1 mg ml^−1^) showed higher values than that of ZnO only or compared to other TCNE concentrations (0.5, and 2 mg ml^−1^). These *μ*_e_ values are undeniable in demonstrating the improvement in *J*_sc_ and FF of iOSCs. The ETLs type and electron only device structure is shown in Table 4.

**Table 4:** Summarizing the electron mobility values of fabricated devices with ZnO and different TCNE concentrations coated on ZnO as ETLs

Device type ITO/ETLs/PTB7:PC71BM/LiF/Al	Electron mobility [cm^2^ V^−1^ s^−1^]
Pristine ZnO	6.23 × 10^−4^
ZnO/**TCNE** (**0.5 mg ml**^**−1**^)	1.28 × 10^−3^
ZnO/**TCNE** (**1 mg ml**^**−1**^)	1.34 × 10^−3^
ZnO/**TCNE** (**2 mg ml**^**−1**^)	5.22 × 10^−4^

The experimental data displayed thus confirmed the importance of an ultra-thin TCNE interlayer sandwiched between the ZnO/photoactive layer to tailor the ZnO surface, resulting in reduced carrier recombination of photogenerated charges, thus advancing the device *J*_sc_ and FF.

**Fig. 10:**
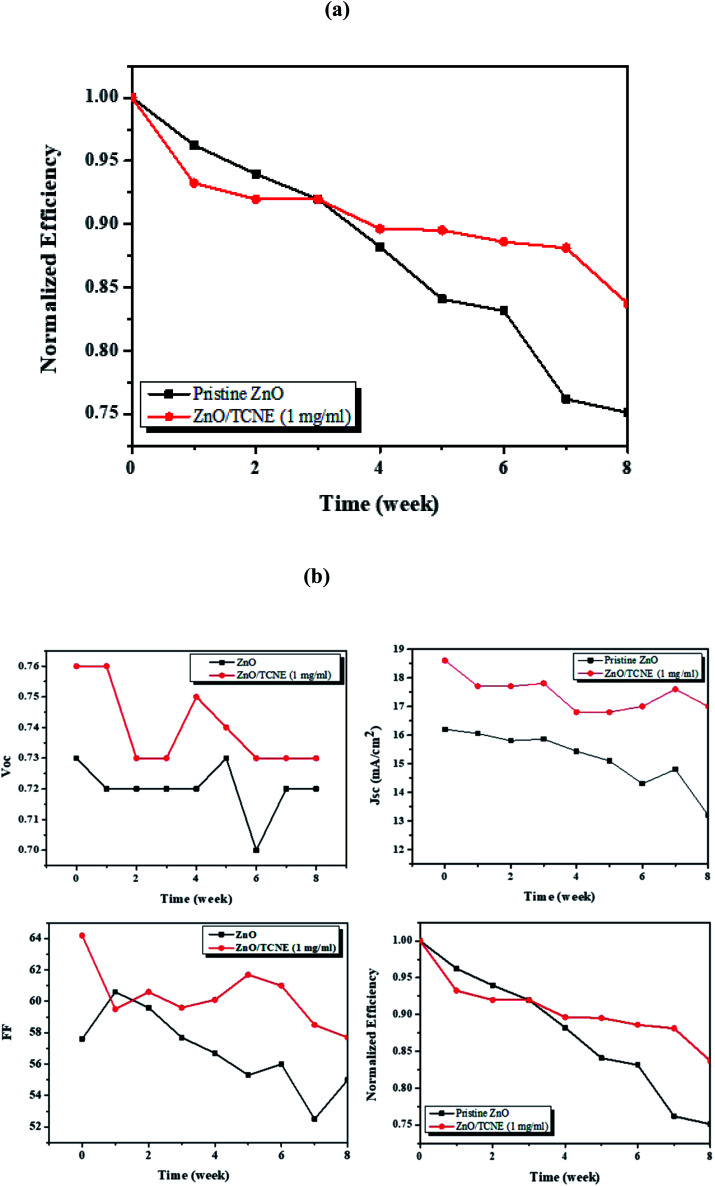
(a) Normalized power conversion efficiencies of ZnO and ZnO/TCNE ETLs, respectively, devices as a function of eight weeks of storage time (b) *I*–*V* parameter breakdown of *V*_oc_, *J*_sc_, FF, and PCE *vs.* time in eight weeks.

Finally, we also investigated the durability of the fabricated reference and modified devices, shown in Fig. 10. The devices were tested periodically for eight weeks. We primarily investigated the stability of ZnO/TCNE devices in an N_2_ filled glove box system along with that of pristine ZnO ETL devices. One can see that the power conversion efficiency of the TCNE-modified device maintained 83.70% after 8 weeks of storage, which is far better than that of the reference device, which is remaining about 72.82% of the original values.

**Fig. 11:**
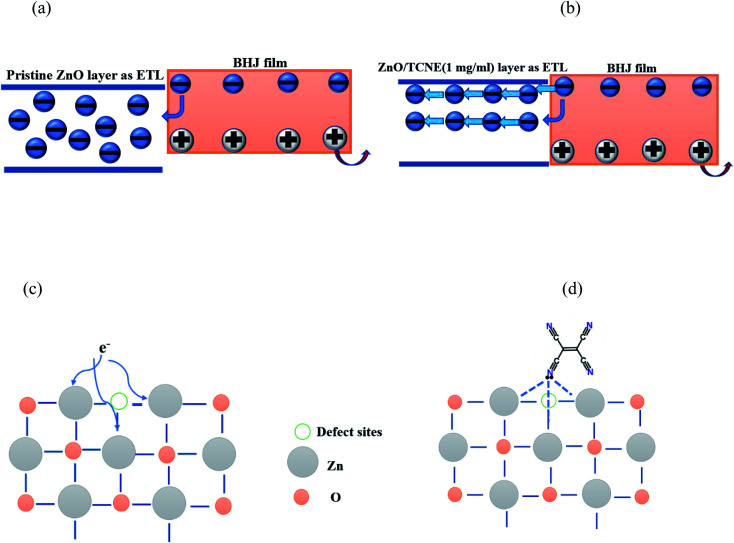
Schematic representation of (a) ZnO and (b) ZnO/TCNE films surface, and illustration of interaction mechanism of (c) ZnO, and (d) ZnO/TCNE films.

Due to the best performance and longer lifetime of ZnO/TCNE based devices, it is interesting to show a model for the interaction between TCNE and ZnO surface, as shown in Fig. 11. Fig. 11a displayed that the electron might be trapped in the V_O_ oxygen vacancies (defect sites) in the electron extraction process. Fig. 11b showed that the lone pair electron on the nitrogen of TCNE molecule interacted with the partial positive charge on Zn atom beside oxygen vacancies. Meanwhile, it provides the interfacial dipole for the efficient extraction of electrons. The majority of these surface defects are oxygen vacancies (V_O_), making ZnO films susceptible to the adsorption of environmental oxygen and water molecules. TCNE may act as an “oxygen binder”, thus reducing the number of V_O_ in ZnO films surface.^52,53^

Furthermore, the significant improvement in the TCNE modified devices is attributed to the effective passivation of surface defects of ZnO by coating with the ultra-thin TCNE layer.

## 4. Conclusion

In summary, a new surface modifier TCNE was used. We have successfully explained the improvement of interfacial compatibility between the ITO and the BHJ blend film with TCNE modified ZnO, formed a co-interfacial layer of ZnO/TCNE to improve the PCEs in iOSCs. The co-interface layer has higher FF and electron mobility than the pristine ZnO film. By playing with various concentrations, excellent compatibility was attained between the ZnO film and the active layer. Simultaneously, the appropriate morphology of ZnO/TCNE films was generated, promoting the electron extraction and transport from the photoactive layer to the cathode.

Further, the improvement in *J*_sc_ (from 16.2 to18.6 mA cm^−2^) and FF (from 57.6% to 64.2%), resulting in a best-enhanced PCE (from 7.47% to 8.59%), compared to that of without TCNE. Hence, we assume that the study presented here would empower the development of new interfacial modifier for high-performance OSCs and many other optoelectronic device applications. Conclusively, this combination of ease-of-fabrication, low-temperature processing, high device performance, and device flexibility is expected to help push these inverted BHJ OSCs closer to commercial viability.

## Conflicts of interest

There are no conflicts to declare.

## Supplementary Material
